# Understanding Post Entry Sorting of Adenovirus Capsids; A Chance to Change Vaccine Vector Properties

**DOI:** 10.3390/v13071221

**Published:** 2021-06-24

**Authors:** Coralie F. Daussy, Noémie Pied, Harald Wodrich

**Affiliations:** Microbiologie Fondamentale et Pathogénicité, MFP CNRS UMR 5234, University of Bordeaux, 146 rue Leo Saignat, CEDEX, 33076 Bordeaux, France; coralie.daussy@u-bordeaux.fr (C.F.D.); noemie.pied@u-bordeaux.fr (N.P.)

**Keywords:** adenovirus, virus entry, vaccine vector, autophagy, innate immunity, adaptive immunity, intracellular trafficking, SARS-CoV-2

## Abstract

Adenovirus vector-based genetic vaccines have emerged as a powerful strategy against the SARS-CoV-2 health crisis. This success is not unexpected because adenoviruses combine many desirable features of a genetic vaccine. They are highly immunogenic and have a low and well characterized pathogenic profile paired with technological approachability. Ongoing efforts to improve adenovirus-vaccine vectors include the use of rare serotypes and non-human adenoviruses. In this review, we focus on the viral capsid and how the choice of genotypes influences the uptake and subsequent subcellular sorting. We describe how understanding capsid properties, such as stability during the entry process, can change the fate of the entering particles and how this translates into differences in immunity outcomes. We discuss in detail how mutating the membrane lytic capsid protein VI affects species C viruses’ post-entry sorting and briefly discuss if such approaches could have a wider implication in vaccine and/or vector development.

## 1. Introduction

Viral infections, unlike bacterial infections that can be treated with antibiotics, remain a threat to health as very few antiviral remedies exist. Therefore, vaccination is the only widely applicable strategy to protect human (and animal) populations from viral infections. Until recently, vaccination against viral infections was mainly considered in the context of preventing childhood diseases or as the annual “flu” shot for influenza infection prevention. With the outbreak of the SARS-CoV-2 health crisis in 2020, vaccination has returned to the center of public attention as it is now seen as the only sustainable way out of this pandemic. In this context, adenovirus (Ad) vector-based vaccines have emerged as one of two major successful vaccination strategies. Following emergency approval, Ad vaccines are now being injected to a large proportion of the earth’s population in an unprecedented effort to install planetary immunity against SARS-CoV-2.

Adenovirus vector vaccines were not widely used before the COVID-19 outbreak, but their success is not unexpected. Viruses are in general very suitable vaccine platforms. They are biologically optimized and adapted for nucleic acid transport, and they elicit immune responses. Adenoviruses have many features that make them particularly ideal vaccine candidates. They have a low and well-characterized pathogenic profile paired with a high infectivity. They are technologically approachable and have been extensively used for vectorization. Their high production yield and stability allow industrial production under good manufacturing practice (GMP) conditions and provide practical features (i.e., storage) for regional distribution at a reduced cost [1]. Furthermore, Ad cell delivery into antigen-presenting cells is efficient, and they naturally provoke an innate immune response that acts as an adjuvant to boost vaccination success [2,3].

Understanding Ad biology has been crucial in developing Ad-based (vaccine) vectors. For example, the use of non-human or rare Ad serotypes in some of the currently marketed SARS-CoV-2 vaccines was deliberately chosen to prevent pre-existing vector immunity from reducing vaccine efficiency. The use of viral gene-deleted vectors provides improved vector safety. However, there are still many aspects of the viral life cycle that have not been considered when developing Ad-based vaccine vectors, and knowledge on rare or non-human genotypes is limited, including the ones currently used as SARS-CoV-2 vaccines. With this review, we would like to raise awareness for potential modifications that could be exploited when developing Ad vectors for medical purposes including vaccination. We focus on the viral capsid and how the choice of genotypes influences the uptake und subsequent subcellular sorting. We describe how understanding capsid properties such as stability during the entry process can change the fate of the entering particles and what different immunity outcomes are observed. As an example of how capsid stability and fate can be changed, we discuss in detail how mutating the membrane lytic capsid protein VI affects species C viruses. Finally, we briefly discuss if such approaches have the potential to be exploited for vaccine and/or vector development.

### 1.1. Adenoviruses Constitute a Diverse Family of Infectious Pathogens

Historically, Ad was first isolated in 1953 from adenoids of patients with respiratory infections [4], and soon after, the term "adenovirus" was universally adopted [5]. The family of *Adenoviridae* has more than 120 members, divided into five genera—depending on whether they infect mammals (e.g., *Mastadenoviridae*), birds (e.g., *Aviadenoviridae*), reptiles (e.g., *Atadenoviridae*), amphibians (*Siadenoviridae*), or fish (*Ichtadenoviridae*) [6,7]. The genus *Mastadenoviridae* also accounts for more than 70 types of human Ads [8,9], which are classified into seven species (A–G), according to their morphological, biological, and physicochemical properties, as listed in Table 1 [10,11]. Furthermore, Ads can also spontaneously recombine, thereby generating new species [12], as it was the case for AdE4, which is the only human species E virus and is likely to have resulted from the recombination between human species B and a simian Ad [13]. Although infections in humans with non-human Ads are naturally rare, they are not impossible [14,15]. The capacity of a variety of non-human Ads to infect (or transduce) human cells makes them attractive (vaccine) vector platforms. Among them are Ads derived from chimpanzees [16,17,18], gorilla [19], sheep [20], cow [21], dog [21,22], or the new world monkey [23].

Adenovirus infections can cause different pathologies depending on their cellular or tissue tropism including respiratory, ocular, urinary, or gastrointestinal diseases (see Table 1). Tissue tropism is often the result of distinct receptor usage by different Ads and may also be related to intrinsic particle stability [24]. Most infections with Ads are mild or asymptomatic in an immune-competent host. Primary Ad infections are mostly occurring during childhood and result in strong protective immunity. In contrast, in immune-suppressed individuals, Ads can cause uncontrolled, severe, and life-threatening systemic infections leading to serious cell toxicity, which can result in excessive inflammation and multiple organ failure, causing high mortality [12]. The best-studied human Ads are the species C viruses type 2 and 5, which are predominantly found in patients with upper respiratory tract and gastrointestinal infections [25] and which will be in the major focus of this review. Adenoviruses can be responsible for occasional outbreaks, such as species B viruses causing pneumonia (e.g., Ad3, 7, and 14) among immune-competent individuals [26] or Ad40/41 of species F and Ad12, 18, and 31 of species A, which infect the gastrointestinal tract (as reviewed in [26,27]). Species D viruses are a very diverse group and prone to recombination and are a leading cause of eye infections [28].

### 1.2. Adenovirus Capsids Are Metastable Structures

Despite their diversity, all Ads follow a similar structural and organizational framework. Adenoviruses are non-enveloped DNA viruses containing a relatively large, 26–45 kb linear double-stranded DNA genome [29,30,31]. The capsid is ~70–90 nm in diameter and has the form of an icosahedron with 20 facets and 12 vertices [32]. The capsid consists of three major structural proteins (hexon, penton, and fiber) and a variety of minor cement proteins (i.e., IIIa, VI, VIII, and IX in species C viruses) that stabilize the structure. The high-resolution structure of several Ad capsids has been obtained by cryo-electron microscopy and X-ray crystallography [24,33,34,35,36]. The icosahedral capsid has a pseudo T25 structure with 720 hexons assembled in different trimers depending on their location in the capsid. The 20 facets of the capsid are composed of nine assembled hexons (so called GON for Group of Nine), whereas at the 12 vertices, hexons are assembled in GOS (for Group of Six) (see [37,38] for more details). The pentameric penton base is inserted into the 12 vertices and serves as the basis for the protruding trimeric fiber [39,40]. The interaction between major and minor capsid proteins stabilizes the capsid, mainly through the interaction with hexons. For example, GON are stabilized by IX [41]; IX and IIIa are themselves stabilized by their interaction with VIII, which links them with GOS and GON, respectively [37]. VIII molecules outline the GON and help to stabilize hexons on the inside of the capsid. Protein VI also stabilizes hexon by binding the hexon trimer central cavity exposed to the inner viral lumen [42,43,44].

The capsid surrounds and protects the viral core composed of the viral genome, the viral core proteins (i.e., V, VII, Mu in species C viruses), and the terminal protein (TP), which is covalently bound to each 5’ end of the Ad genome. Minor capsid proteins interact with core proteins establishing a connection between capsid and genome; however, their spatial organization in relation to the core proteins remains poorly understood. It has been shown that V maintains DNA inside the capsid by interacting with VI (capsid side) and VII (genome side) [45,46,47] and probably contributes to genome release at the nuclear pore [48]. VII is the main capsid protein associated with viral DNA and promotes its condensation inside the capsid [49,50,51]. VII also protects the viral genome from immune detection through cellular factors [50,52,53,54]. Interestingly, capsid protein IX and protein V are only present in *Mastadenoviridae* [55,56], and viral capsids can be produced without IX [57] or protein VII [58], showing some flexibility in the capsid structure.

The genome of human Ads encodes ~45 proteins (including the structural proteins), organized into temporally regulated transcription units [59]. The transcription factor E1A is the first expressed protein and is necessary to initiate viral gene expression [60]. The genome also harbors a 5’ encapsidation sequence and inverted terminal repeats (ITR) at each extremity which are required for replication [59,61,62,63,64]. Adenoviruses encode an adenoviral protease (AVP) which is packed into the particle and processes several proteins (i.e., IIIa, VI, VII, VIII, µ, TP, and 52.55K in species C viruses) [65,66,67]. This step is required for the formation of mature infectious particles and induces the formation of metastable (i.e., less stable) capsids primed for capsid disassembly upon cell attachment and entry [68,69].

There are also species-specific differences in the structure of human Ads, e.g., in the fiber length and flexibility, which directly affects receptor binding [70,71,72]. Differences can also be observed in penton assembly. Penton of species B viruses can form inter-penton contacts, resulting in alternative and less stable virus-like particles that are devoid of genomes and occasionally without fibers. Such particles are called dodecahedron and are studied for the cell transfer of plasmids or peptides as an alternative to Ad vectors. Dodecahedrons better expose a RGD short peptide motif present in penton of most Ad species, which is required to bind target cells [73,74]. Differences in capsid stability, as discussed below, may be an important property that determines the entry fate of Ad particles.

### 1.3. Adenoviruses Follow a Lytic Life Cycle

**Figure 1:**
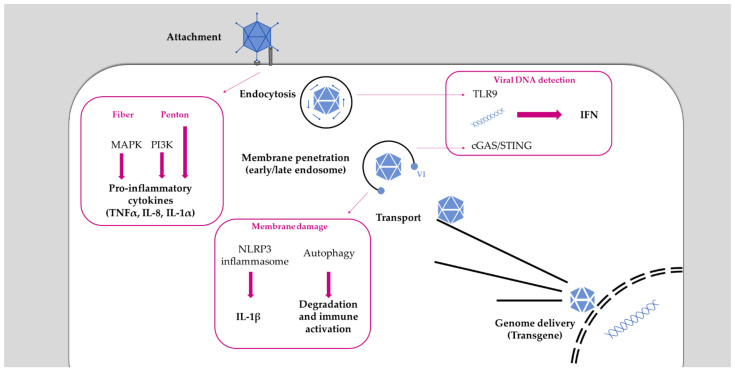
Adenovirus cycle and associated immune response. Along its journey to the nucleus, Ad faces several cellular immune restrictions. Interaction of fiber and penton are required for initial attachment of Ad to cellular receptors; this interaction also induces a signaling cascade leading to the production of pro-inflammatory cytokines. Following entry, Ad is trapped inside an endosome where viral genomes can be detected via TLR9. The membrane lytic function of the viral protein VI allows viral penetration of the endosomal membrane, permitting viral escape into the cytosol. Endosomal escape occurs from within early or late endosome, depending on the Ad species. Membrane penetration of the viral capsid allows exposure of viral DNA in the cytosol that can be sensed by cGAS/STING. Membrane damage is also detected as a danger signal and induces both inflammasome and autophagy activation.

The Ad infection cycle has been extensively reviewed elsewhere [75,76,77]. Here, we briefly focus on the entry part of the life cycle, and, unless stated otherwise, we will refer to species C viruses, which are the best studied (Figure 1). Adenoviruses use the capsid fiber as the primary cell attachment molecule. Most Ads, including species C viruses, use the coxsackievirus and Ad receptor (CAR) to bind target cells [78,79,80,81]. In contrast, some species B viruses preferentially bind the CD46 receptor [82] or desmoglein 2 [83], and some species D viruses were shown to interact with sialic acids [84,85] or directly with αvβ integrins (see Table 1) [86]. Thus, fiber switching between species is an attractive strategy to change tropism [87,88,89]. The primary role of the fiber molecule is to mediate the physical association with target cells, which is followed by a second interaction between penton and integrins, such as αvβ5 [90]. This interaction is mediated by the RGD peptide motif in the penton sequence [91]. Absence of this motif, as in species F viruses, is associated with less efficient cell entry [92]. Integrin binding results in integrin clustering at the cell surface [93] that triggers a signaling cascade, leading to the reorganization of the actin cytoskeleton [94,95] and the endocytic uptake via clathrin-mediated endocytosis or micropinocytosis [96,97]. RGD binding may also destabilize the capsid [98] by decreasing contacts between penton and hexon, helping the capsid to uncoat [99]. As a consequence, Ad particles lose their fiber, and one can find dissociated hexon and penton inside the endosome [100,101,102]. The endosome is a dynamic organelle primed to mature into degradative lysosomes, and Ads have to escape rapidly into the cytosol. Endosome acidification may accelerate the escape process by further weakening the capsid [100,103]. The endosome penetration process relies on the release of the internal capsid protein VI [103,104]. The released VI is characterized by a N-terminal amphipathic helix that binds to the inner leaflet of the endosome and induces positive membrane curvature, resulting in locally confined membrane rupture [75,103,105,106]. Cells respond to the virus-induced membrane rupture and activate a localized autophagy response to clear the damaged organelle [107,108,109]. To avoid degradation, Ads stall autophagy via a short PPxY peptide motif in protein VI until the particle has reached the safety of the cytosol [104,108]. Species C viruses were shown to escape from early endosomes [110]. Other species including A, B, and D first traffic to the lysosome before escaping into the cytosol [111,112,113], which may affect the immune activation triggered by those viruses [114].

Once Ads have reached the cytosol, they engage with dynein motors and use retrograde transport along microtubules to reach the microtubule organizing center [115,116,117]. Motor binding is probably mediated by hexon [115] and may require acidic priming [118]. In the vicinity of the nucleus, capsids switch transport directionality, probably by binding to kinesin motor proteins, and accumulate at the nuclear envelope [115,117,119,120]. They dock at the nuclear pore complex, then capsids are completely disassembled, and genomes are released and imported into the nucleus [51,121,122,123,124]. Once inside the nucleus, the cycle continues by transcription activation of the genome and expression of the immediate early E1A gene, which serves as a transcription factor for all other early transcription units [60,125,126,127,128,129,130]. Following expression of the replication enzymes, viral genomes are replicated and accumulate at the periphery of replication centers [131]. Then, structural proteins are expressed from the late expression unit under the control of the major late promoter [132,133]. Expression of late genes culminates in production and nuclear accumulation of all structural proteins where they assemble into progeny and package newly synthesized genomes. Nuclear assembly of the next generation of Ads then results in particle egress and cell lysis [42,134,135,136]. As mentioned above, assembly of the capsids is followed by maturation, i.e., the proteolytic cleavage of several capsid proteins by the AVP to produce infectious particles [66]. The importance of this process is exemplified by the temperature-sensitive mutant, *Ts*1, initially identified in a mutagenesis screen in Ad2. At the non-permissive temperature, this mutant fails to package the AVP, and assembled particles do not undergo maturation, resulting in hyperstable particles [137]. The responsible AVP mutation was genetically introduced into Ad5 retaining this phenotype. *Ts1* capsids are still able to attach to cell receptors and to be endocytosed, but then remain trapped in the endosome due to their failure to liberate protein VI [103,138,139]. 

## 2. Immune Detection of Adenovirus

Adenoviruses are widespread, and a large majority of the population has been confronted with this virus, mainly during childhood. Adenoviruses are highly immunogenic, and every encounter provokes the development of specific anti-Ad immunity, both innate and adaptive, that can last a lifetime. This is important in the fight against the virus and renders Ads less harmful to the immune-competent host, but it can be an obstacle when Ads are to be used as a vector in vaccinology or gene therapy [140,141]. Understanding how Ad (and its vector derivatives) activates the immune system will allow us to improve the effectiveness and safety of vaccines. Observations in vivo, from patients participating in clinical trials, clearly showed that Ad-based vectors have the ability to induce potent immune activation [142,143], sometimes with fatal consequences [144]. In addition, various studies carried out in vitro or in animal models [145] suggest a key role of the viral particle itself (i.e., capsid and incorporated DNA) in activating the initial immune response. Here, we briefly focus on immune responses associated with the entering capsid; for a more in-depth overview, see previously published reviews [146,147,148,149].

### 2.1. Adenoviruses Trigger Cell Intrinsic Immunity

With the development of Ads as vectors, it became apparent that cell intrinsic or innate immunity induced by Ads is a response to the invading virion. Interestingly, both replicative and non-replicative viruses (i.e., UV-inactivated virus, non-replicative vectors, or empty capsids) activate immune responses in the infected cells, showing that the capsid has a key role in this response [145,150,151,152,153,154,155,156].

In general, Ads are sensed by cells at several steps throughout the entire viral life cycle and activate innate immunity pathways (Figure 1). This activation starts with the Ad fiber binding to the cell surface receptor CAR, which activates the mitogen-activated protein kinase (MAPK) signaling that leads to the activation of the transcription factor NF-κB. This trigger results in the production of pro-inflammatory cytokines (IL-6, IL-8) [157,158]. However, depending on Ad species, other receptors usage may modulate the strength of the signaling cascade involved in cytokine expression [159]. The interaction between viral penton and cellular integrin is a further trigger of immune activation. A study in murine macrophages showed that this binding activates the expression of IL-1α via the RGD motif [160]. This might be restricted to immune cells because in other cell models (e.g., HeLa, epithelial cells line), the role of the integrin/RGD interaction in chemokines and pro-inflammatory cytokines production was not confirmed [151,161,162]. This suggests a more important role of the internalization process itself in immune activation than the interaction with integrins. Indeed, penton binding to integrin also activates the phosphoinositide 3-kinase (PI3K) signaling to increase viral internalization [163]. Moreover, PI3K activation has been shown upon Ad infection to trigger the production of pro-inflammatory cytokines, such as TNFα [164], and the internalization process has been described as required for immune activation [165]. These studies suggest that immune activation mainly occurs at a post-internalization step, highlighting the importance of post-entry sorting (reviewed in [166]).

Once in the endosome, Ads are exposed to intraluminal pathogen-recognition receptors (PRR) such as TLR9, which is a double-stranded DNA sensor restricted to immune cells. Murine macrophages can sense Ad vectors via TLR9 to induce the production of pro-inflammatory cytokines [167]. TLR9 was also strictly required for IFNα/β production when murine plasmacytoid dentritic cells (pDC) were challenged with Ad [168]. However, in TLR9 knock-out mouse models, IFNα/β was still produced, suggesting that, in cells other than pDC, IFN production occurs independently from TLR9 signaling [168,169].

Viral genomes (or vector genomes) become accessible to sensing during or after the endosomal escape of the virus. Once they reach the cytosol, they are sensed by the cytosolic double-stranded DNA sensor cGAS [168,170]. Upon viral genome recognition, cGAS promotes phosphorylation and nuclear translocation of the transcription factor IRF3 to drive IFNα/β expression, inducing an antiviral state. Endosomal passage requires virus-induced endosomal membrane damage to facilitate endosomal escape of viral particles. Membrane rupture thus considerably contributes to immune activation, highlighted by the fact that the escape defective *Ts1* mutant fails to activate a complete and efficient immune response [161,162,170,171]. A pivotal role in the inflammatory response upon Ad membrane penetration is played by the tank-binding kinase, TBK1, suggested to be part of a down-stream cytosolic sensing of the viral genome via the cGAS/STING pathway [168,170].

Adenovirus membrane damage also results in the release of cathepsin B from the endo-lysosomal compartment, causing oxidative stress, which activates the NLRP3 inflammasome, resulting in IL-1β maturation [114]. Species C viruses escape from early endosomes, while species B viruses traffic until late endosomes, presumably due to differences in receptor usage. Acidification in late endosome/lysosome is more likely to activate lysosomal acid hydrolases. Both species induce inflammasome activation [172], but, probably owed to the residing time in the endosome, the extent of their responses differ. Globally, species B viruses induce a stronger innate and adaptive immune activation than species C viruses [113,173,174]. This may explain why species B viruses are more pathogenic, causing outbreaks in immune competent hosts [26]. It further suggests that cells are able to discriminate the penetration compartment (i.e., endosome vs. lysosome, [109]) and adapt the efficiency of immunity. Endosomal membrane damage during the Ad escape process can also be a danger signal in itself. As discussed in detail below, membrane damage results in the cytosolic exposure of intraluminal glycans, which are detected by the cell as danger signals (as reviewed in [109]). The detection of exposed glycans by galectins subsequently activates autophagy, triggering a second branch of antiviral immunity [108].

### 2.2. Adenoviruses Provoke an Aadaptive Immune Response and Subvert Antiviral Autophagy upon Cell Entry

Innate immune responses provoked by Ads or their vectors subsequently translates into adaptive immunity through chemokine secretion that attracts immune cells (neutrophils, natural killer cells, and macrophages) and reinforces potent antigen presentation [153,154]. Adenoviruses are highly immunogenic, and their main immunogens are the major capsid proteins (hexon, penton, and fiber) that trigger the species-specific production of neutralizing antibodies by B cells [175,176,177]. Neutralizing antibodies are mostly directed against the hyper-variable loops of the hexon protein [178,179,180] and represent a major limitation for the use of Ads as vectors, if derived from the same serotype. This problem has been circumvented by the use of less seroprevalent or non-human Ads [181,182,183,184,185]. Despite this anti-vector immunity, when Ads are used as vaccine platforms (e.g., to elicit immune protection against Ebola in clinical trials), they still induce a strong B cell response, allowing the production of antibodies against the desired vaccine immunogen that can last for up to 6 months [186]. In addition, pre-existing vector immunity could be overcome with higher vaccine vector doses, as in the case of the SARS-CoV-2 vaccine vector based on the highly seroprevalant AdC5 [187].

As with neutralizing antibodies, Ad infections trigger a persistent T-cell immunity, mainly through CD4+ activation. This protection can still be found in adults, suggesting a long-lasting immunity from childhood [188,189,190]. In addition, activation of CD8+ is also described to maintain a cytotoxic response [191,192,193]. This T-cell activation is not necessarily serotype-specific and allows cross-protection against different Ad subgroups and serotypes [192,193,194]. Moreover, in stem-cell-transplanted patients infected by Ad, a strong activation of CD4+ and CD8+ has been described [195]. This activation is directed against hexon peptides and leads to a clearance of Ad viremia, emphasizing the major role of both CD4+ and CD8+ T-cell activation in immune protection. While this T-cell-based immunity is directed against the Ad particle, Ads also trigger strong cellular immunity based on the T-cell response against the vaccine antigen, which is an advantage for long-lasting immunity when using recombinant Ad vectors as vaccine platforms [196,197]. Importantly, data from clinical trials have confirmed that when Ad is used as a vaccine platform (with the desired antigen as transgene), it induces both cytotoxic CD8+ T-cell and CD4+ T-cell activation, leading to memory immunity against the expressed transgene [184,186,198,199,200,201,202].

**Figure 2:**
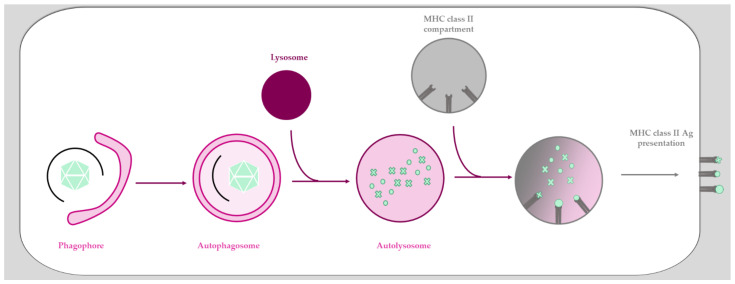
MHC class II presentation by autophagy. Autophagy starts with the formation of a double membrane structure called phagophore that will elongate around the cargo to form a double-membrane vesicle called autophagosome. After fusion with lysosome, the cargo will be degraded in a so-called autolysosome. Further fusion of this vesicle with MHC class II compartment allows the loading of peptides for the ensuing MHC class II antigen presentation.

T-cell activation relies on the presentation of viral peptides through the major histocompatibility complex (MHC). Briefly, antigen presentation by MHC class I results from proteasomal degradation, and the peptides obtained will induce CD8+ T-cell activation, allowing priming and proliferation of a cytotoxic T-cell response. MHC class II antigen presentation results from lysosomal proteolysis, and the resulting peptides will induce CD4+ T-cell activation upon presentation, triggering the subsequent activation of B cells [203]. It is now well characterized that autophagy promotes peptide presentation to the MHC class II molecules [204]. Not surprisingly, autophagy is one of the oldest defense mechanisms against infection and an important part of the cellular repertoire against invading pathogens, participating in both innate and adaptive immune responses. It can directly sequester a pathogen for degradation (xenophagy) and participates in their presentation to the immune system [205,206,207]. Macroautophagy (more commonly called autophagy) is a conserved lysosomal degradation pathway that participates in many fundamental physiological processes such as homeostasis and innate and adaptive immunity [208,209,210]. Autophagy is characterized by the formation of a double membrane vesicle called “autophagosome” that engulfs cytoplasmic cargoes (i.e., organelles, aggregates, and pathogens) destined for degradation (Figure 2). During this process, cargo-containing autophagosomes fuse with lysosomes in order to be degraded and recycled.

Antigen presentation via autophagy has been described for both MHC class I and class II [204]. After induction of autophagy, intracellular antigens are engulfed in autophagosomes, fusing with MHC class II containing compartments to provide them with external pathogen material (Figure 2) [211]. The role of autophagy in this particular MHC class II antigen presentation was first described for Epstein-Barr virus infection, where inhibition of autophagy led to decreased CD4+ T-cell activation [212,213]. To date, examples include several viruses including herpes simplex virus [214], influenza virus [215], and human immunodeficiency virus type 1 (HIV-1) [216], and probably extends to Ads (see below). MHC class II presentation is consistent with the process of autophagy as it is a cytosolic defense mechanism.Autophagy is also involved in MHC class II cross-presentation of extracellular antigens [214]. Indeed, the MHC presentation of extracellular ovalbumin is more efficient when the autophagy machinery (and especially ATG5) is functional, and the uptake of extracellular material was shown to involve a non-canonical form of autophagy called LC3-associated phagocytosis (LAP) [217]. Once present in the LAP-osome, extracellular antigens can be exposed through MHC class II presentation [218]. Autophagy is also involved to some degree in MHC class I presentation, although the vast majority of the epitopes are presented via a mechanism strictly dependent on the transporter associated with the antigen processing (TAP) complex [219]. TAP-independent presentation mediated by autophagy was so far shown only under TAP depletion conditions [219,220]. If this mode of presentation truly exists remains controversial [221] and is discussed elsewhere [204,222,223].

To prevent MHC presentations, Ads dedicate a great deal of their genome, namely the E3 region, to undermine the MHC presentation system in cells [224,225,226,227,228]. In most (vaccine) vectors, these genes are absent, and recent work suggests that increasing antigen presentation by MHC could improve Ad vaccine efficiency [229,230]. Autophagy plays a crucial role in processing Ad antigens for MHC class II presentation in Ad-induced immunity [108,229].

Montespan et al. showed that Ads induce autophagy upon endosome penetration and that entering particles cleared by autophagy in this process are efficiently presented to the immune system [108]. This observation is consistent with the study published by Klein et al. showing that in the context of oncolytic Ads, the viral antigens are matured by a JNK-dependent form of autophagy [229]. However, autophagy in the context of cancer plays a dual role. As a homeostasis keeper, autophagy limits tumor initiation [231] while in advanced cancer autophagy acts as a survival process to favor tumor growth [232,233]. Thus, in the context of oncolytic vectors, tumor-specific autophagy features should be carefully evaluated. 

## 3. Adenoviruses as (Vaccine) Vectors

Vectorization of a virus involves the exploitation of its natural properties, while simultaneously minimizing the associated biological risk. In these terms, Ads are excellent vector candidates for vaccine development [234]. Adenovirus vectors are stable, are easy to produce to a high titer, and have large cloning capacities. This allows insertion of a transgene, i.e., to express an antigen of choice. They have a broad infectivity spectrum and transduce non-dividing cells, and their genome is non-integrative [1]. As described above, they also provoke an inflammatory response in antigen-presenting cells (APCs) that serves an adjuvant function to amplify the immune response necessary to vaccinate efficiently [2,3,142,235,236]. Moreover, Ad-derived vectors have been extensively used in preclinical and clinical trials to prove their safety [237,238]. To eliminate the unwanted part of the replication cycle, viral replication needs to be suppressed to prevent cell lysis and viral propagation. Adenovirus vector development started during the early 1990s as a platform for a large range of therapeutic approaches, such as gene therapy, oncolytic vectors, and vaccine development. Most of the early vector developments were modified versions of species C viruses.

### 3.1. Adenovirus Vector Development, a Generational Approach

The first generation of an Ad vector was made by deleting the E1 and E3 region from the viral genome, notably encoding the immediate early transcription factor, preventing these vectors from replicating. These vectors had a cloning capacity of around 8 kb to insert a transgene with regulatory sequences [239,240,241]. The first-generation vectors were produced in human embryonic kidney (HEK) cells stably transformed with a part of the Ad5 viral genome that complements the E1 region in *trans* [242,243,244,245,246,247]. One of the major drawbacks of this system is that it allows the reconstitution of replication-competent genomes after spontaneous recombination [248]. The additional deletion of E3, which is not necessary for vector production, increases the size of the transgene cassette [243], and promotes a better immune response [240]. Unfortunately, first-generation Ad vectors were not completely transcriptionally inert and still elicited a strong immune response against viral capsid proteins that were expressed at baseline levels [141,249,250]. This anti-vector immunity contributed to vector toxicity and short-term elimination of transduced cells [145]. The second generation of Ad vectors additionally had the E2 and E4 regions removed from the vector genome. E2 encodes the replication enzymes and E4 additional regulatory proteins [251,252,253]. This allowed space for 10.5 kb of transgene sequences, including up to four expression cassettes [254]. Likewise, the supplemental deletions strongly reduced unwanted recombination and prolonged transgene expression [255]. This was probably the result of a reduced aberrant viral gene expression that further reduces anti-vector immunity [141,250,251]. Adenoviral vectors of the third generation are conceptually different. They consist of a packaged vector genome and a non-packaged viral genome (helper virus) that produces vector particles. Such vectors are also known as “helper-dependent”, “gutless”, or “high-capacity” (HC-Ad) vectors. They have the highest possible cloning capacity and can carry sequences of up to 36 kb [256,257,258]. High-capacity Ads lack any viral coding sequence except ITRs and a short encapsidation signal that permits genome packaging and which is absent from the helper virus [259,260,261,262]. This strategy has allowed us to finally overcome most anti-vector immunity, permitting the long-term expression of transgenes [263,264,265,266,267]. However, HC-Ads are not part of the current vaccine vector repertoire.

### 3.2. Adenovirus as a Vaccine Vector

Before exploring how Ad vectors have been exploited, it is noteworthy to mention that vaccination against Ad species B viruses type 4 and type 7 themselves was developed in the early 1970s [268]. This oral live vaccine is mandatory since 2011 for military recruits in the USA and dramatically decreased infection rates, reaching virtually zero [269,270,271]. This highlighted the intrinsic efficiency and safety of Ad as a vaccine and opened the field to use Ad as a vaccination platform. To date, over 200 Ad-based vaccines entered clinical trials [272]. Many of them are directed against infectious pathogens such as HIV-1 and Ebola [230,273,274]. Adenovirus-based vaccines are not without drawbacks. A recently developed anti-HIV vaccine, based on the species C virus Ad5, used a mixture of vectors expressing the HIV-1 gag, pol, and nef genes [275,276]. The vectors were safe, immunogenic, and well-tolerated. Unfortunately, the resulting STEP clinical trial had to be prematurely stopped [277,278,279]. Not only has this vaccine been shown not to protect against HIV infection, but epidemiological data suggested that vaccination with Ad in this case increased the HIV infection risk [280,281,282]. Having a pre-existing immunity against Ad5 was identified as a potential post-vaccination risk factor [283,284]. However, this observation was not confirmed in similar studies [285,286] and remains mechanistically unclear [287,288]. Next to HIV, Ad-based vector vaccines have been developed in the fight against Ebola (Ad-EBOV), a recurrent threat in Western Africa [289]. All Ad-EBOV vaccines use the Ebola glycoprotein (GP) as the vaccine antigen. Initial preclinical trials in rodents and primates used Ad5 and showed protection over 3 months with production of neutralizing antibodies and CD8+ activation [273,290,291,292]. This vaccine went through phase II clinical trial; however, pre-existing immunity to Ad5 in the vaccinated population has led to variation in the level of elicited neutralizing antibodies [293,294,295,296,297]. To overcome this issue, vaccines based on the less prevalent species D Ad26 have been developed [186,298,299]. However, Ad26 is less immunogenic in humans, resulting in decreased vaccination efficiency, and required a prime boost using a measles virus-based vaccine (MVA). This vaccination regime has reached phase III clinical trials [289,300,301]. Use of non-human Ad vectors has also been under development [184]. Use of chimpanzee ChAd3 as a vaccine platform against EBOV showed protection in rodents and in chimpanzees [302,303], and clinical trials employing those vectors are at different steps of progress [17,300,301,304,305,306]. Until recently, HIV-1 and EBOV vaccines were the two main examples for the application of Ad vector-based vaccines, but they are not the only ones. Different attempts have been made to employ Ad vaccine vectors against various pathogens such as *Mycobacterium tuberculosis* [307,308,309,310], dengue virus [311,312,313], hepatitis B and C [314,315,316,317,318,319,320], rabies [321,322,323,324], or influenza [325,326,327,328]. However, their efficacy was always below expectations (for more details, see [18,142,300,329]). The EBOV vaccine candidate has remained the only practically applied Ad vaccine and is used with some restrictions in the Democratic Republic of Congo. This vaccine is based on the Ad26 type (Ad26-ZEBOV/MVA-BN-FILO), and, as of May 2020, 20,339 people received the first dose of vaccination, and 9560 were fully vaccinated [230,301,330].

Adenovirus-vectorized vaccines have received a major public boost in response to the SARS-CoV-2 pandemic. The urgency of the situation has made it possible to capitalize on their development, including experience gained with vaccines raised against coronaviruses that caused previous regionally confined SARS-CoV-1 outbreaks [331]. The coronavirus spike (S) glycoprotein was identified as the best vaccine antigen for immunity to coronavirus infections [332,333]. To date, four Ad vector vaccines, all encoding the spike antigen, are in an advanced state and have been granted for emergency use in large parts of the world. The applied vector strategies have been slightly different, but essentially involve the use of first-generation vectors. They range from using species C Ad5-based vectors (Ad5-nCoV), overcoming pre-existing immunity with a high vector dose (CanSino) [187,334], to the use of less prevalent species D virus Ad26, using a stabilized spike glycoprotein (*JNJ-78436735/Ad26.COV2.S*) [335]. An alternative approach uses ChAdOx1-S-(AZD1222), an Ad vector from chimpanzees [336,337]. The only vaccine in use based on a heterologous prime-boost strategy to overcome vector immunity (*Gam-COVID-Vac/Sputnik V*) applies first an Ad26-based vector and then in the booster injection uses an Ad5 vector [338,339]. In all cases, clinical trial data have been very promising, showing high levels of induced protective immunity, and emergency use has been granted in several countries for the use of Ad-based vaccines. Furthermore, epidemiological surveys in the ongoing vaccination campaigns in different countries suggest an overwhelming success in the use of Ad-based SARS-CoV-2 vaccines.

## 4. Modulating Adenovirus (Vaccine) Vector Efficacy, the Capsid Leads the Way

Adenovirus (vaccine) vector design has to take into account a number of factors. These include maintaining a long and sustained expression of the encoded antigen and the activation of an immunological context (adjuvant effect) that permits efficient translation of the antigen expression into a persistent adaptive immunity directed against the immunogen but not against the vector itself. The immunological context is provided by the innate immune activation that the vaccine vector elicits during administration. In this context, the efficiency with which innate immune sensors detect the vector is important, and will in turn determine the amplitude of the adaptive response. As described in the sections above, innate immune sensing is mainly taking place at the post-entry level, concomitant with Ad endosome penetration. The efficient expression of the (vaccine) antigen depends then on the balance between the level of vector clearance vs. successful delivery and expression of vector genomes to the nucleus. With the development of capsid display vectors, expression of the transgene is not required to elicit immune activation. This strategy is based on the insertion of an epitope of interest into Ad capsid proteins, allowing its direct exposure to the immune system. Even if this approach has some advantages, most vaccine vectors rely on genetically encoded genes. For further reading on capsid display vectors, please see these reviews, [340,341].

Pre-existing immunity against a viral vector (e.g., 30–90% against the species C virus Ad5, depending on the geographic location) can reduce vector uptake and decrease the antigen expression and the resulting immune response [342,343,344]. Several strategies have been developed to decrease anti-vector immunity and have been recently reviewed [249]. Briefly, the neutralizing antibodies mainly recognize the hypervariable loop of hexons and as a consequence are serotype-specific [178,179,180]. These hypervariable loops can therefore be replaced by those of another, less prevalent serotype [178,345]. This prevents the vector from being neutralized and inactivated. As Ad5 is the most seroprevalent type, using rare serotypes such as Ad26, Ad35, or non-human Ads would also avoid this issue [177,346,347,348,349,350]. However, a major drawback with the use of rare types is their lower immunogenicity that makes them less efficient regarding vaccine development [298]. Therefore, strategies to decrease anti-vector immunity should be developed in parallel to strategies to enhance vector immunogenicity in order to obtain the most efficient vector. Even if the initial level of neutralizing antibodies against the vector is low, vaccination with a given vector type may trigger a response that prevents its future use. Priming of the anti-vector response also takes place at the post-entry level, when vector particles are sorted and processed as antigens themselves. Eliciting an immunologically favorable context for vaccination success may come at the expense of increased priming for vector immunity. Understanding the parameters that determine the fate of Ad vectors after uptake may provide an opportunity for vector design.

### 4.1. Stability Lies in the Species

Receptor-mediated uptake and escape from the endo-lysosomal compartment are hallmarks of Ad infection and vector transduction. The initial uptake is determined by the interaction of the virus fiber and penton molecules with cell receptors. Fiber and penton are common to all Ads, but, according to their species, they engage with different cellular receptors (Table 1). Species C viruses use CAR, and entering particles escape from an early endosomal compartment [79]. If species C viruses (Ad5) are engineered to contain a fiber molecule from a species B virus (Ad16), the resulting particle (Ad5F16) then escapes from a lysosomal compartment (as shown by costaining with the lysosomal marker LAMP1) instead of the early endosome [351]. This delayed escape from lysosomes is a feature shared with all tested species B viruses [111,352,353]. Furthermore, the hybrid Ad5F16 as well as species B viruses elicit a stronger pro-inflammatory response than species C viruses [159,351]. This is attributed to increased detection via intraluminal TLR9 in immune cells [354]. In addition, lysosomal membrane damage, unlike early endosome damage, causes strong oxidative stress. Lysosome rupture co-releases lysosomal hydrolases such as cathepsin B that can damage mitochondria, thus amplifying the damage response through inflammasome activation [355,356,357]. The Ad uptake process destabilizes the capsid, allowing the release of the viral protein VI [103,106]. Released protein VI then penetrates the membrane from within the endo-lysosomal compartment to allow the virus to gain access to the cytosol. Comparing species C and B shows that both viruses are equally efficient in cytosol entry and genome delivery, but differ in their escape kinetics [111]. The fact that a simple fiber swap can transfer the entry and immunogenic properties from species B to a species C virus is remarkable. It provides a proof of concept that hybrid vectors with specific properties can be designed. The underlying difference seems to be that protein VI release is delayed in species B viruses (or the hybrid virus) and may require either acidification or another, unknown disassembly trigger. Since protein VI release is delayed in this case, this could reflect an increased capsid stability. Indeed, species C Ad5 viruses were shown to release the fiber molecule upon cell binding, a step assumed to weaken the capsid and prime the disassembly process for protein VI release. In this context, fiberless Ad5 were shown to be less stable than native particles [358]. In contrast, non-matured, hyperstable *Ts1* Ad2/5 do not release fiber or protein VI and are poor inducers of inflammation, since they are neither triggering TLR9 nor causing membrane damage [114,138,359,360]. Whether fiber swapping with species B prevents initial fiber release and renders the species C capsid more stable has not been investigated but is probable. Capsid stability maybe an essential determinant of post-entry capsid fate and, by extension, a feature that could be exploited for vector design. Species B viruses may be more stable due to the use of a different receptor or through a unique fiber-penton interaction. In contrast, species F viruses Ad40/41 infect the gastrointestinal tract and have increased capsid stability due to their tropism and their adaptation to the harsh gut environment [24,361]. The gut has an intrinsic temperature that is a few degrees above the upper respiratory tract targeted by species C viruses (37 °C vs. 33 °C) [362]. Thus, Ad40/41 are intrinsically more thermostable, supporting the infection process [24], making them also difficult to propagate [24,361,363]. In addition, they lack a RGD motif in the penton molecule, resulting in a further reduced cell entry [92]. Whether these unique capsid properties translate into escape from a lysosomal compartment or partial disassembly/protein VI release defects upon entry is not known. In contrast, these properties may provide a context for the development of oral vaccine applications. Unlike species B fibers, species F fiber-harboring vectors have a decreased immune activation [364] when applied at standard environmental conditions. If a given human Ad species is now taken out of context and employed as a vaccine vector, it could be worthwhile to look at the natural tropism of this vector to look for clues as to predict its behavior. The currently used vaccine vector based on Ad26 is a species D virus, and its natural tropism is the eyes and the gastrointestinal tract [365]. Species D viruses including Ad26 are less immunogenic than Ad5 [366] and were shown to traffic to the late endosomal compartment, probably involving delayed protein VI release. Consequently, blocking acidification of the late endosomal compartment further reduced its immunogenicity [113]. In contrast to human Ads, the knowledge on non-human Ads also used in vaccination and vectorization is very limited. Chimpanzee-derived Ads including the ChAd3 are closely related to species D viruses [367,368]. Interestingly, Ad5 vectors are more immunogenic than chimpanzee-derived Ad vectors and have a higher transduction potential. This observation was not linked to differences in the penton RGD motif or the fiber, and its mechanistic reason remains unknown [369]. Potential differences in capsid stability have not been investigated. Another non-human Ad exploited as a vector is the canine Ad type 2 (CAV-2). This vector has the remarkable property to transduce neurons selectively, but not surrounding glia cells, unlike an Ad5 control vector, despite both vectors using the same receptor [370,371,372]. This ability may be linked to an increased capsid stability, allowing uncoating only in neuronal cells. Dogs have a body temperature that is ~2 °C higher than humans, which potentially results in elevated CAV-2 capsid stability [373], analogous to the species F viruses. CAV-2 poorly elicits an innate immune response, consistent with a lack of efficient disassembly in target cells [372]. Thus, CAV-2 vectors might be another example where a biophysical property linked to its natural tropism or host has the potential to provide novel vector properties. 

### 4.2. Fine-Tuning the Capsid Structure, the Example of Protein VI in Species C Ad2/5

**Figure 3:**
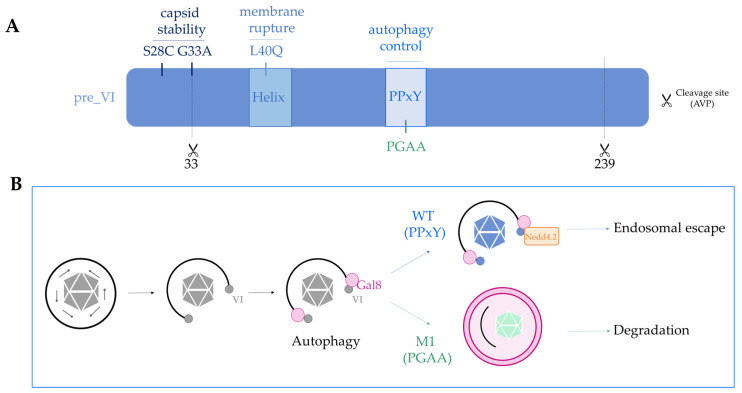
Protein VI, a multifunctional viral protein determining the post-entry fate of the particle. (**A**) Domain organization of the membrane lytic protein VI. Protein VI is produced as a precursor form (pre_VI) and processed by the viral protease (AVP) at two sites. A role for the N-terminal part in capsid stability has been shown by two-point mutation (S28C and G33A). An amphipathic helix is responsible for the membrane lytic activity of protein VI, and a point mutation in this domain (L40Q) reduces the lytic activity. Protein VI encodes a conserved PPxY motif required for viral control of the cellular autophagy response against membrane penetration. Mutating the motif (PGAA) reduces viral infectivity. (**B**) Adenovirus membrane penetration via protein VI is recognized by the cell through glycan binding galectin 8 followed by autophagy induction. WT virus uses the PPxY motif in protein VI to recruit the ubiquitin ligase NEDD4.2 to suppress or halt autophagy until endosomal escape is completed. A mutation in this motif (PGAA) abolishes viral autophagy control and renders the mutant virus (M1) particle susceptible to autophagic degradation and enhanced antigenic presentation.

If capsid stability and protein VI release determine the downstream fate of the viral capsid, the question arises whether this can be modified in Ad vectors, i.e., to attribute novel properties to viral vectors (Figure 3). Most of our understanding of how Ads penetrate the endo-lysosomal compartment comes from the species C viruses Ad2/5. The discovery of protein VI as a membrane lytic factor was essential to understand this process [103,106]. Protein VI encodes a N-terminal amphipathic helix that is the actual membrane lytic part of the protein. During virus production, protein VI is expressed as a precursor protein that associates with hexon trimers via the amphipathic helix [42,374]. The complex is imported into the nucleus via transport signals encoded in the N- and C-terminus of protein VI, which are removed during virus assembly and maturation by the viral protease. The C-terminal 11 amino acid peptide also activates the protease, providing a smart way to link virus assembly with shielding of the amphipathic helix. The *Ts1* Ad2/5 mutant virus has a packaging defect for the protease, and none of the capsid proteins, including protein VI, is processed. Resulting particles are hyperstable, retain fibers, and do not deploy protein VI. As consequence, *Ts1* particles are sorted into lysosomes upon receptor-mediated endocytosis. The exact location of protein VI in the mature virus is not clear, but the cleaved N-terminus localizes to the inner hexon surface of peripentonal hexons [37,104,375]. Two point mutations in protein VI, G33A and S28C, were shown to increase the Ad5 capsid stability (Figure 3A) [376,377]. The first mutant precedes the N-terminal protease cleavage site in protein VI and impairs the processing. Resulting particles are partially immature and are characterized by an endosome lysis defect, suggesting that the N-terminus of protein VI stabilizes intra-virion protein-protein interactions [376]. The second mutant was chosen for its ability to form intra-molecular disulfide bridges [377]. The virus contained partially unprocessed proteins, potentially including protein VII, but surprisingly did not show any infectivity defects, despite its increased stability. Whether either of the viruses has an altered immune activation profile is not known. Another point mutation in protein VI that affects the virus fate is the protein VI L40Q mutation, directly situated in the amphipathic helix (Figure 3A) [378,379]. Viruses with this mutation mature normally but are partially defective for the endosome lysis step. In contrast to the above mentioned mutants, L40Q virions are less stable than their wild type counterparts. Instead of a delay, protein VI as well as penton are released prematurely from the L40Q capsid. L40Q virions are less infectious and subject to partial lysosome sorting. However, the L40Q mutation does not completely abolish the membrane lysis, and some viruses escape to the cytosol, resulting in an overall ~four-fold reduced infectivity of a L40Q vector [379,380]. The observations suggest that the amphipathic helix contributes with its interaction to the particle stability. It is not known if and how the L40Q mutation alters the immunogenic property of Ad5 vectors. Recent observations suggest that release of protein VI from the capsid is triggered by internal competition with protein VII for the same or overlapping binding sites in hexons [375]. Consistently, a mutant virus devoid of protein VII fails to escape from the endosome and has a defect in endosome lysis [58]. Further understanding the structural and dynamic organization of the internal capsid (i.e., protein VI) and core proteins (i.e., protein V and VII) in the particle and during cell entry will greatly help to understand what determines (reversible) capsid stability.

Controlling the efficiency of immune activation of Ad vectors is a major interest for the development of vaccines, and expanding MHC presentation of antigens would improve vaccine development [381]. As mentioned previously, autophagy regulates immune activation and feeds peptides to the MHC. Autophagy is one of the oldest cellular defense mechanisms against infection, yet Ads, as adapted human pathogens, have not evolved mechanisms to avoid autophagy upon entry. Instead, they seem to embrace autophagy activation and to divert the response to promote infection. Protein VI plays a central role in controlling the autophagy response of the cell. Adenovirus membrane lysis by protein VI leads to the recruitment of galectin 3 and galectin 8, which are recognized by the cell as danger signals (Figure 3B) [108,109,382]. Detection of Ad penetration sites by galectins activates selective autophagy in the infected cell, as illustrated by the recruitment of autophagy receptor (e.g., NDP52 andp62) and LC3 punctae formation [108]. Interestingly, Ad5 vector particles manage to escape from the ruptured endosome and are able to traffic to the nucleus. Inhibiting autophagy does not affect viral infectivity, but delays their genome delivery to the nucleus, showing that Ads hijack the cellular machinery for their own profit [108]. Escape from the endosome therefore allows the virus to avoid degradation by autophagy via an active process. This process was discovered by yet another protein VI mutant, the M1 mutant. All human Ads (and several non-human Ads) encode a highly conserved PPxY peptide motif in protein VI. In the M1 mutant, this motif was changed to PGAA (Figure 3) [104]. Both wild type and the M1 mutant efficiently release protein VI upon entry and trigger autophagy after rupture of the endosomal membrane. However, the M1 mutant virus is unable to escape efficiently into the cytosol, and EM images have shown that M1 mutant viruses are trapped in ruptured endosomes, targeted by autophagy. Consequently, the M1 mutant virus was shown to have a strong infectivity defect, which is restored upon the pharmacological and genetic inhibition of autophagy. The study showed that wild type viruses use their PPxY motif to recruit the ubiquitin ligase NEDD4.2 and limit the maturation of autophagosomes long enough to escape to the cytosol. Furthermore, this property allows the virus to limit its antigenic presentation [108]. Importantly, the M1 mutant vector injected into mice resulted in much stronger anti-vector immunity and a reduced CD8+ response to the encoded transgene, showing that a simple capsid alteration can have a profound impact on the immunogenicity of the Ad5 capsid. Whether any of the other protein VI mutations affects immunogenicity is not known. However, the presence of the PPxY motif in the L40Q virus may explain why the vector retains a relatively high infectivity despite the membrane lysis defect [379,380]. Because the endosome lysis is less efficient, and L40Q vectors end up in part in the lysosomal compartment, it is conceivable that the elicited immune response is yet different from either wild type, *Ts1*, or M1 Ad5 capsids.

## 5. Conclusions

As demonstrated in this review, development of Ad-based (vaccine) vectors with specific and desired properties is in part driven by a comparison between available Ad species without necessarily understanding the mechanistics behind existing differences. The lack of data concerning the underlying biology for most vectors, other than species C, means that relying on rare or non-human serotypes bears a risk of missing out on properties or overlooking problems that can arise. We strongly advocate to ameliorate this lack of knowledge by pairing vector development and application with investigating the underlying vector and virus biology. In the meantime, we believe that the existing knowledge base on Ad biology, especially for species C viruses, could help with the design of new Ad vectors, including rare or non-human species, using rational design. In this review, we highlighted the link between Ad post-entry sorting and the ensuing innate immune response and provided examples of how Ad post-entry sorting differs between species and has been modulated. The non-exhaustive list of possible capsid modifications exemplified on capsid protein VI and restricted to Ad2/5 shows the great potential that still lies buried in the Ad capsid, waiting to be exploited. Lysosomal membrane damage, such as early endosomal membrane damage, also triggers autophagy, but in addition results in oxidative stress that alters the innate immune response of the cell [383]. Altering the endo-lysosomal escape process of a species B, species D, or non-human Ad by introducing desirable mutations into the respective protein VI gene is only one of the possible options that come to mind. Other capsid proteins may hold additional functions related to transport or immune modulation. Extending rational capsid modifications to last-generation HC-Ad vectors and optimized antigen expression cassettes hold great promise for the next generation of Ad-based vaccine vectors. 

## Figures and Tables

**Table 1:** A simplified overview of human Ads and non-human Ads and their uses for vaccination purposes. Species of human Ads, their receptor, and main tropism.

Species	Type	Tropism	Receptor	Remarks
A	12, 18 and 31	Intestinal	CAR	
B	B1 : 3, 7, 16, 21 and 50 B2 : 11, 14, 34 and 35	Respiratory and ocular	CD46/DSG-2	
C	1, 2, 5 and 6	Respiratory	CAR	Ad5 : used as vaccine against SARS-CoV-2 and in clinical trial against Ebola and HIV-1 (but prematurely stopped)
D	8-10, 13, 15, 17, 19, 20, 22-30, 32, 33, 36-39, 42-49	Ocular and intestinal	CAR	Ad26 : used vaccine against Ebola and SARS-CoV-2
E	4	Respiratory and ocular	CAR	
F	40,41	Intestinal	CAR	Oral vaccine
G	52	Intestinal	CAR/sialic acid	
Non-human (Chimpanzee) (Canine)	ChAd3 ChAdOx1 CAV-2	—	CAR	ChAd3 : used as a vaccine in a clinical trial against Ebola ChAdOx1 : used as a vaccine against SARS-CoV-2 CAV-2 : can target neuronal cells
